# Factors predicting recanalization following stent-assisted coil embolization of unruptured intracranial aneurysms with long-term follow-up

**DOI:** 10.3389/fneur.2024.1351940

**Published:** 2024-04-24

**Authors:** Yu Deok Won, Young Deok Kim, Seung Pil Ban, O-Ki Kwon

**Affiliations:** ^1^Department of Neurosurgery, Hanyang University Guri Hospital, Guri, Gyonggi-do, Republic of Korea; ^2^Department of Neurosurgery, Seoul National University Bundang Hospital, Seoul National University College of Medicine, Seongnam, Republic of Korea

**Keywords:** intracranial aneurysm, endovascular procedure, recanalization, stent-assisted coil embolization, long-term follow-up

## Abstract

**Objective:**

Stents have been widely used for coil embolization for intracranial aneurysms. Few studies have analyzed the risk factors of recanalization through long-term follow-up observation of only stent-assisted coiling. We analyzed the risk factors for recanalization through long-term observations.

**Methods:**

A total number of 399 unruptured aneurysms treated by stent-assisted coil embolization between 2003 and 2016 in a single institution were analyzed for determining the factors associated with recanalization including the patient characteristics, aneurysms, and procedural variables. All patients underwent angiographic follow-up with digital subtraction angiography or magnetic resonance angiography at 24 months or more following the procedure.

**Results:**

Recanalization occurred in 8%. The mean time for the recanalization was 21.1 ± 14.0 months (range, 5–51 months). The receiver operating characteristic curve analysis indicated areas under the curve for a maximum aneurysm size of 0.773 (cut-off, 6.415 mm). Multivariate analysis revealed that the maximum aneurysm size and parent artery curvature at which the aneurysm developed were significantly associated with recanalization. In parent artery curvature, the bifurcation group (OR, 9.02; 95% CI, 2.53–32.13; *p* = 0.001) and the convex group (OR, 3.68; 95% CI, 1.17–11.50; *p* = 0.025) were independent predictors of recanalization compared with the straight group.

**Conclusion:**

The maximum aneurysm size and parent artery curvature are risk factors associated with recanalization in stent-assisted coil embolization.

## Introduction

Coil embolization has increased proportionately compared to microsurgical clipping in the treatment of intracranial aneurysms. The use of stents is one of attributable factors. Stents have been thought to play an important role in wide neck aneurysm treatment while increasing the rate of complete occlusion and lowering the rate of recanalization (1). However, recanalization still arises even with the use of a stent. The recanalization of intracranial aneurysms diminishes the therapeutic efficacy of coil embolization, thereby increasing the risk of retreatment or bleeding.

There are few studies on the risk factor analysis of recanalization for stent-assisted coiling only. For long-term follow-up studies, the average follow-up period for most studies was less than 20 months (2–4). Due to the increasing use of stents, it is necessary to analyze the risk factors of recanalization through long-term follow-up observation of stent-assisted coiling. In this study, we conducted a long-term analysis of patients undergoing stent-assisted coiling in a single center to determine the risk factors of recanalization.

## Methods

### Patient selection

**Figure 1:**
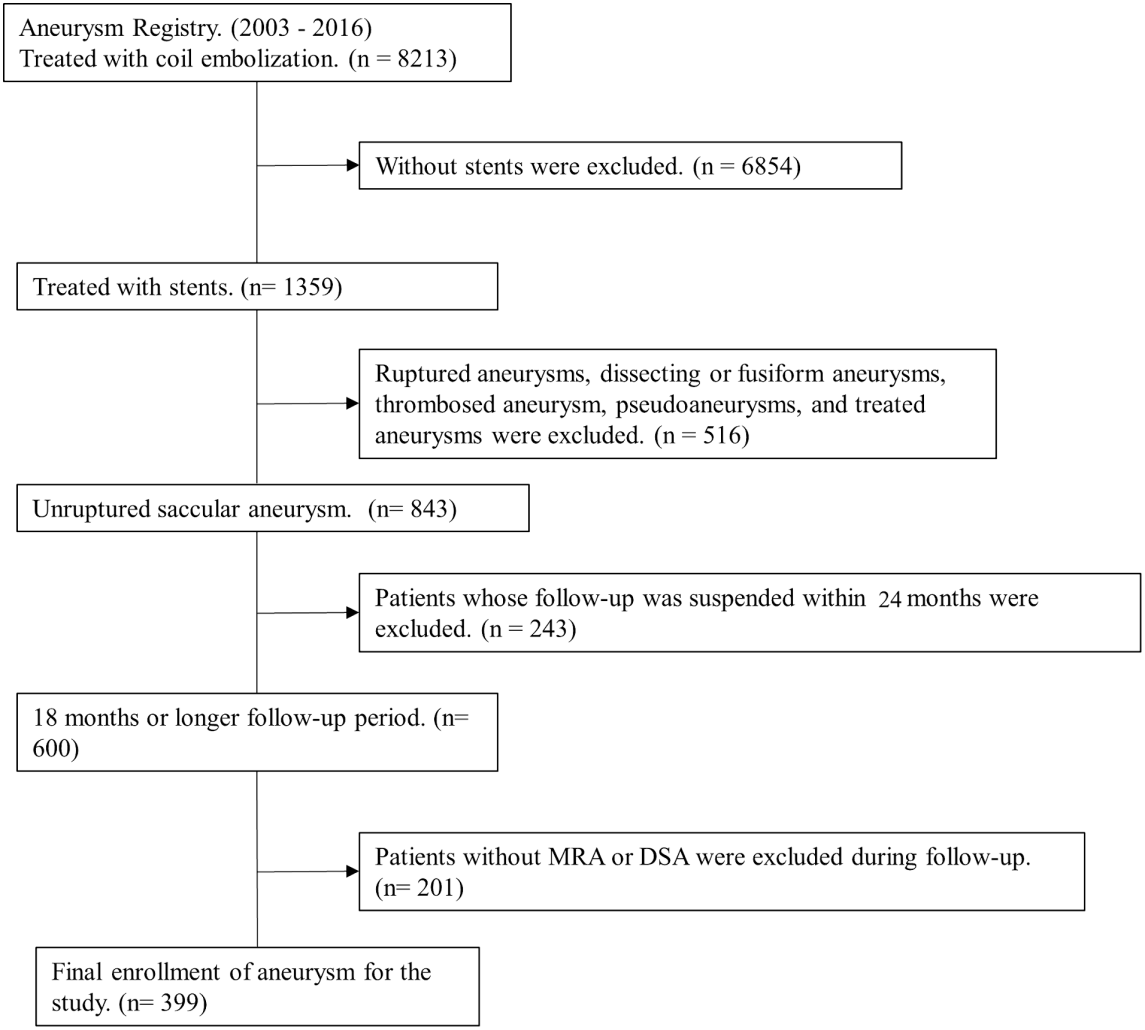
Flow chart of the patient selection.

We collected data from our aneurysm registry for 8,213 patients treated using coil embolization between January 2003 and December 2016. Inclusion criteria for patient were as follows: (1) stent used, (2) unruptured saccular aneurysms, (3) 24 months or longer follow-up period, and (4) follow-up imaging studies with MRA or DSA (Figure 1). Finally, 399 unruptured saccular aneurysms in 399 patients were included and analyzed.

This study was approved by the Institutional Review Board of our hospital and conformed to the tenets of the Declaration of Helsinki. Owing to the retrospective nature of the study, informed consent was waived.

### Definition of angiographic features

DSA was performed in all patients prior to coil embolization. The aneurysm size was measured by two neurosurgeons using three-dimensional (3D) imaging with VitreaWorkstationTM (Vital Images Incorporated, Minnetonka, MN, USA). The maximum aneurysm size was noted as the longest part of the aneurysm except for neck diameter. The aspect ratio is the ratio of the maximum length perpendicular to the neck to the neck diameter. The dome neck ratio is the ratio of the maximum length horizontal to the neck to the neck diameter. The packing density was calculated using a software from AngioCalc.^1^

**Figure 2:**
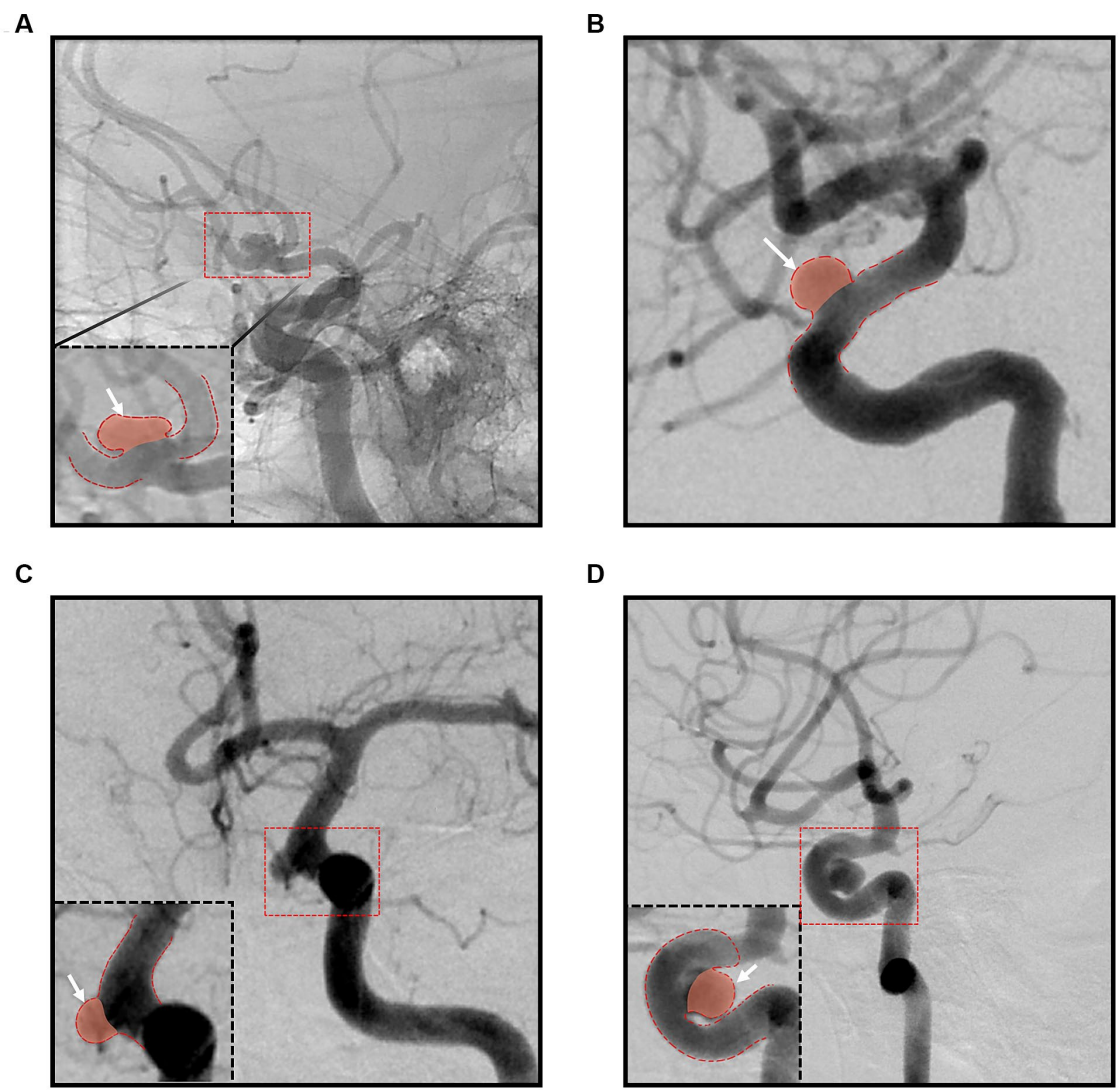
Classification according to the parent artery curvature; **(A)** bifurcation group; **(B)** straight group; **(C)** convex group; **(D)** concave group.

The aneurysm types were categorized as bifurcation and sidewall; aneurysms in places where the diameters of the bifurcation vessels were not significantly different were considered as bifurcation aneurysms, whereas branches of the parent artery that were smaller than the parent artery or an absence of branch artery were considered sidewall aneurysms. The parent artery curvature was classified into bifurcation, convex, concave, and straight based on the relationship between the aneurysm and the parent artery, using an image with a more pronounced aneurysm neck among the working angle images at the time of the coil embolization (Figure 2). In sidewall aneurysms, the aneurysm was classified as ‘convex’ and ‘concave’ in the convex and concave part of the parent artery, respectively, and ‘straight’ when the parent artery was not in either direction. The degree of coil packing was classified as complete neck, residual neck, and residual aneurysm using the Raymond-Roy occlusion classification (5).

### Endovascular stent-assisted coil embolization

All procedures were elective and performed by two experienced neurosurgeons specializing in neuroendovascular treatment under general anesthesia. All patients were administered standard dual antiplatelet preparation that included 100 mg of aspirin and 75 mg of clopidogrel daily for 5 days prior to the procedure. One day before the procedure, the platelet function was checked by VerifyNow (Accumetrics, San Diego, CA, USA). High on-treatment platelet reactivity (HTPR) was defined as a P2Y12 reaction units value of 220 or more based on a previous study (6). Patients with HTPR were administered a modified antiplatelet preparation based on a previously reported study (7). During the procedure, IV heparin was administered, a bolus dose of 3,000 IU was administered immediately following femoral arterial sheath insertion, and subsequently 1,000 IU was administered every hour.

All patients underwent stent-assisted coil embolization and the most commonly used stent was the Enterprise stent (Codman, Raynham, Massachusetts, USA). Since a single stent was utilized in all cases, there was no potential confusion arising from the use of multiple stents in flow diversion. Following the procedure, dual antiplatelet treatment was continued for a year. Thereafter, 100 mg of aspirin was administered for an additional year. Aspirin administration was further prolonged in certain patients based on their medical conditions including atherosclerosis.

### Imaging follow-up and radiologic outcome

Recanalization of the aneurysm was defined as the compaction of the coil mass or revisualization of the aneurysm with the growth. This included both major recurrences that required additional surgical treatment and minor recurrences that were kept under close observation.

All patients underwent MRA every 6 months for 2 years after the coil embolization, followed by every 1 year. If recanalization was suspected in the MRA scan, a DSA was performed for confirmation. The other patients in whom recanalization was not suspected in the MRA scans were consistently followed up for MRA or DSA.

### Statistical methods

Continuous variables are expressed as the mean ± standard deviation, while discrete variables are expressed as a count with percentage. The Chi-square test and Student’s t-test were used to assess the clinical differences between the two groups, which were classified based on the presence/absence of recanalization.

To determine the optimal cut-off value of the aneurysm size that may be associated with recanalization, the receiver operating characteristic (ROC) curve was used with the dependent variable of the recanalization.

Uni- and multivariate logistic regression analyses were used to assess the risk factors for recanalization of the aneurysm and to calculate the OR based on the different predictive factors.

All statistical analyses were performed using R version 3.3.3.^2^

## Results

A total of 399 aneurysms in 399 consecutive patients (mean age 56.0 y; 78.9% female) who were treated at our hospital with unruptured intracranial aneurysms between 2003 and 2016 were analyzed. The aneurysm size was 6.6 ± 3.6 mm (range, 2.4–28.2 mm) and the neck diameter was 4.5 ± 1.9 mm (range, 1.7–13.8 mm). The mean follow-up period was 49.9 ± 20.4 months (range, 24–155 months). The predictive factors were analyzed between the two groups (‘patients with no recanalization’ versus ‘patients with recanalization’) under variables including sex, age, presence of hypertension, diabetes mellitus, smoking, aneurysm location, type, size, height, neck diameter, aspect ratio, dome neck ratio, packing density, stent type, parent artery curvature, Raymond-Roy occlusion classification, and complications (Tables 1, 2).

**Table 1:** Clinical characteristics and the results of the procedure and complications of patient.

Variables	No recanalization (*N* = 367)	Recanalization (*N* = 32)	*p*
Clinical variables			
Female, n (%)	289 (78.7)	26 (81.2)	0.915
Age, years, mean ± SD	55.6 ± 10.5	60.3 ± 10.3	0.016
Hypertension, *n* (%)	145 (39.5)	21 (65.6)	0.007
Diabetes, *n* (%)	36 (9.8)	4 (12.5)	0.858
Smoking, *n* (%)	58 (15.8)	3 (9.4)	0.476
Packing density, mean ± SD (%)	28.2 ± 7.0	24.4 ± 7.3	0.003
Stent type, *n* (%)			0.592
Enterprise	305 (83.1)	28 (87.5)	
LVIS	30 (8.2)	1 (3.1)	
Neuroform	32 (8.7)	3 (9.4)	
Parent artery curvature, *n* (%)			<0.001
Bifurcation	52 (14.2)	10 (31.2)	
Convex	76 (20.7)	14 (43.8)	
Concave	33 (9.0)	2 (6.2)	
Straight	206 (56.1)	6 (18.8)	
Raymond-Roy occlusion classification, *n* (%)			0.539
Complete	107 (29.2)	8 (25.0)	
Residual neck	158 (43.1)	17 (53.1)	
Residual aneurysm	102 (27.8)	7 (21.9)	
Complications, *n* (%)			
Intraoperative	2 (0.5)	1 (3.1)	0.580
Postoperative	9 (2.5)	1 (3.1)	1.000

SD, standard deviation.

**Table 2:** Aneurysm characteristics.

Variables	Patients with no recanalization (*N* = 367)	Patients with recanalization (*N* = 32)	*p*
Location, *n* (%)			0.002
Anterior	327 (89.1)	30 (93.8)	
Cavernous ICA	7 (1.9)	4 (12.5)	
OphA	36 (9.8)	3 (9.4)	
SHA	126 (34.3)	5 (15.6)	
Supraclinoid ICA	93 (25.3)	5 (15.6)	
PCoA	27 (7.4)	5 (15.6)	
AChA	1 (0.3)	0 (0)	
ICA bifurcation	7 (1.9)	0 (0)	
ACoA	16 (4.4)	6 (18.8)	
ACA	5 (1.4)	0 (0)	
MCA bifurcation	9 (2.5)	2 (6.2)	
Posterior	40 (10.9)	2 (6.2)	
Basilar bifurcation	22 (6.0)	2 (6.2)	
SCA	3 (0.8)	0 (0)	
PCA	1 (0.3)	0 (0)	
PICA	1 (0.3)	0 (0)	
VA	13 (3.5)	0 (0)	
Aneurysm type, *n* (%)			0.028
Bifurcation	54 (14.7)	10 (31.2)	
Side wall	313 (85.3)	22 (68.8)	
Maximum aneurysm size, mm, mean ± SD	6.1 ± 2.8	11.4 ± 6.4	< 0.001
Aneurysm height, mm, mean ± SD	4.5 ± 2.3	8.7 ± 5.8	< 0.001
Aneurysm neck diameter, mm, mean ± SD	4.3 ± 1.7	6.6 ± 2.6	< 0.001
Aspect ratio, mean ± SD	1.1 ± 0.4	1.3 ± 0.8	0.059
Dome neck ratio, mean ± SD	1.4 ± 0.4	1.7 ± 0.7	0.040

OphA, ophthalmic artery; SHA, superior hypophyseal artery; PCoA, posterior communicating artery; AChA, anterior choroidal artery; ACoA, anterior communicating artery; ACA, anterior cerebral artery; SCA, superior cerebellar artery; PCA, posterior cerebral artery; VA, vertebral artery; SD, standard deviation.

Recanalization occurred in 32 aneurysm (8%) and the mean interval from the initial treatment to recanalization was 21.1 ± 14.0 months (range, 5–51 months). Intraoperative complications developed in three cases (thrombus, 2; vasospasm, 1). Postoperative complications, which were defined as complications occurring postoperatively up to the last follow-up, occurred in 10 cases (transient ischemic attack, 3; infarction, 5; cranial nerve palsy, 2).

The procedural data demonstrated that a lower packing density and parent artery curvature were associated with recanalization. The mean packing density of patients with recanalization was 24.4 ± 7.3% compared to 28.2 ± 7.0% in those without recanalization (*p* = 0.003). A significant difference was observed in the parent artery curvature between the groups. In the recanalization group, 31.2 and 43.8% were bifurcation and convex, respectively while in the non-recanalization group, 14.2 and 20.7% were bifurcation and convex, respectively. There was no significant difference noted among the stent type. There were no significant differences in the Raymond-Roy occlusion classifications and in the complications between the groups.

### Risk factor analysis of recanalization

In the univariate analysis, age, hypertension, maximum aneurysm size, aspect ratio, packing density, and parent artery curvature were significant factors for recanalization. In the multivariate analysis, maximum aneurysm size (OR, 1.31; 95% CI, 1.16–1.49; *p* ≤ 0.001) and parent artery curvature were significantly associated with recanalization (Table 3). In parent artery curvature, the bifurcation group (OR, 9.02; 95% CI, 2.53–32.13; *p* = 0.001) and the convex group (OR, 3.68; 95% CI, 1.17–11.50; *p* = 0.025) were independent predictors of recanalization compared with the straight group.

**Table 3:** Univariate and multivariate logistic regression analyses of recanalization after stent assisted coil embolization based on predictive factors.

	Univariate		Multivariate	
Variable	OR (95% CI)	*p*	OR (95% CI)	*p*
Sex				
Female	Reference		Reference	
Male	0.86 (0.34–2.15)	0.739	1.08 (0.30–3.92)	0.908
Age (per 1-year increase)	1.05 (1.01–1.09)	0.017	1.03 (0.98–1.08)	0.251
Hypertension				
No	Reference		Reference	
Yes	2.92 (1.37–6.24)	0.006	2.01 (0.75–5.37)	0.165
Diabetes				
No	Reference		Reference	
Yes	1.31 (0.44–3.96)	0.628	0.52 (0.13–2.07)	0.353
Smoking				
No	Reference		Reference	
Yes	0.55 (0.16–1.87)	0.339	0.48 (0.07–3.15)	0.445
Aneurysm location				
Anterior circulation	Reference		Reference	
Posterior circulation	0.55 (0.13–2.37)	0.418	0.18 (0.03–1.17)	0.072
Maximum aneurysm size (per 1-mm increase)	1.28 (1.18–1.39)	<0.001	1.31 (1.16–1.49)	<0.001
Aspect ratio (per 1 increase)	2.68 (1.44–4.96)	0.002	0.92 (0.35–2.46)	0.871
Packing density (per 1-percentage increase)	0.93 (0.88–0.98)	0.004	1.01 (0.94–1.08)	0.785
Stent type				
Enterprise	Reference		Reference	
LVIS	0.36 (0.05–2.76)	0.328	0.47 (0.05–4.34)	0.505
Neuroform	1.02 (0.29–3.55)	0.974	0.94 (0.18–4.80)	0.941
Parent artery curvature				
Straight	Reference		Reference	
Bifurcation	6.60 (2.30–19.00)	<0.001	9.02 (2.53–32.13)	0.001
Convex	6.33 (2.35–17.05)	<0.001	3.68 (1.17–11.50)	0.025
Concave	2.08 (0.40–10.75)	0.382	0.76 (0.07–7.83)	0.817
Raymond-Roy occlusion classification				
Complete	Reference		Reference	
Residual neck	1.44 (0.60–3.45)	0.415	2.87 (0.93–8.84)	0.066
Residual aneurysm	0.92 (0.32–2.62)	0.873	1.85 (0.49–6.97)	0.365

**Figure 3:**
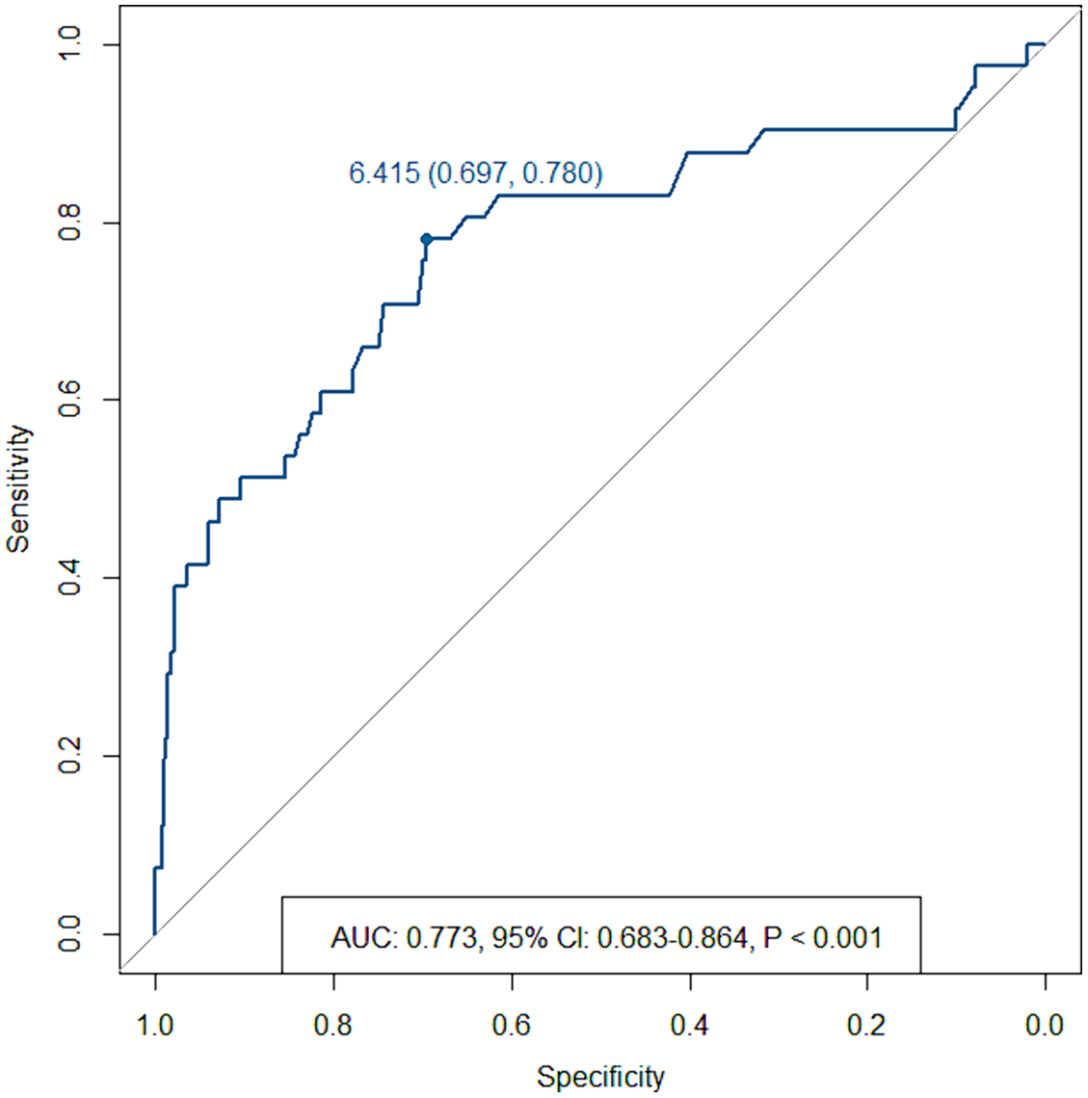
Receiver operating characteristic curve for recanalization based on the maximum aneurysm size.

Since the sample size was large, we believe that the cut-off value of recanalization using the ROC curve was significant. In the ROC curve analysis, the area under the curve for aneurysm size was 0.773 (95% CI, 0.683–0.864; *p* < 0.001; cut-off, 6.415 mm) (Figure 3).

## Discussion

Stent-assisted coil embolization is a safe and effective method for treating intracranial aneurysms. Many studies have showed that the recanalization rate significantly decreased with stent use (8–12). This can be attributed to factors such as more coil packing and flow diversion effect (9, 13). Nevertheless aneurysms treated with stents can also recanalize with time. The duration of long follow-up studies necessary for aneurysms treated by stent-assisted coiling is still not clear. In our study, the follow-up period was longer than in most studies (49.9 ± 20.4 months), and it showed that the recanalization rate was similar to that reported in studies with a relatively short-term follow-up (8, 14–16). This could imply that recanalization occurs in short-term rather than in long-term periods.

The risk factors for recanalization were the aneurysm size and the parent artery curvature at which the stent was placed. Many studies have been conducted on the recanalization factors related to the aneurysm size, including the aneurysm dome, the neck diameter, and the aspect ratio (2, 10, 17–20). In a recent prospective multicenter cohort study, recanalization was also found to be associated with aneurysm size and neck diameter (21). However, the neck diameter of the aneurysm and aspect ratio were not associated with recanalization in our study. This lack of association could be since most of the aneurysms had a large neck diameter; hence, the aspect ratios were in the lower range. Furthermore, the utilization of stents significantly facilitates coil packing within the aneurysm neck, potentially elucidating differences observed from studies not using stent.

Some studies have shown that the packing density and recanalization were not related, (22) while others have shown that the packing density was a risk factor for recanalization (11, 23). Sluzewski et al. (23) demonstrated that no recanalization occurred in aneurysms with a volume less than 600 mm^3^ with a packing density of greater than 24%. In small aneurysms with a volume less than 200 mm^3^, no recanalization occurred when the packing density was greater than 20 percent. In our study, the volumes of most of the aneurysms (375 out of 399) were lesser than 600 mm^3^ and mean packing density was 27.9%. The mean packing density in 32 patients who had recanalization was 24.4%. Since our data were relatively selective in terms of aneurysm volume, the packing density was not considered a significant factor for recanalization in our study.

The relationship between the artery curvature at the aneurysm and recanalization was an important finding observed in our study. Multivariate analysis revealed that the recanalization rate was 9 times higher in cases of bifurcation aneurysms than in sidewall aneurysms at a straight parent artery. When the parent artery was convex at the aneurysm, the recanalization rate was 3.7 times higher than the straight parent artery. This could be attributed to the difference in hemodynamic stress (24–27); the larger the inflow angle, the higher the flow velocity and the greater the wall shear stress causing the aneurysms to grow or rupture (24). The inflow angle is measured as the angle between the flow axis of the parent vessel at the height of the aneurysm neck and the main axis of the aneurysm from the center of the neck to the end of the dome. Similarly, the more convex the relationship between the aneurysm and the parent artery, the greater the inflow angle increasing the hemodynamic stress. Since the aneurysm sac is filled with coils following embolization, the flow velocity does not increase; however, the amount of force transferred towards the aneurysm itself is still present. Therefore, a greater force applied to the aneurysm as the inflow angle increases will result in a better compaction of the coil. When the stent is bent, the cells in the convex part become larger than the other parts, which could reduce the flow diversion effect compared to the straight or concave part. Thus, the flow diversion effect of stents would be weaker in the convex part, thereby increasing the possibility of recanalization.

## Limitations

First, due to the retrospective nature of the treatment aspect of the study, the time between the follow-ups was inconsistent despite designing and implementing a prospective follow-up study. Second, as a single-center study, it did not include a variety of treatment approaches of many different institutes in terms of stent-assisted coiling. However, data consistency and accuracy owing to similar treatment and perioperative protocols could be an advantage in a single-center study. Furthermore, the limited number of recurrent cases hindered us from conducting a subgroup analysis of those who experienced recurrence, and we were unable to analyze the distinction between late and early recanalization. Lastly, TOF sequences of MRA without contrast agent may not have the sensitivity to detect minor changes in cerebral aneurysms following coil embolization, potentially leading to underestimation of the recanalization rate.

## Conclusion

Stent-assisted coil embolization in the treatment of aneurysms is an effective and safe procedure. Long-term follow-up suggests that the use of stents has a low recurrence rate. The maximum aneurysm size and the curvature of the parent artery are risk factors for recanalization.

## Data availability statement

The raw data supporting the conclusions of this article will be made available by the authors, without undue reservation.

## Ethics statement

The studies involving humans were approved by the Institutional Review Board of the Seoul National University Bundang Hospital (IRB number: B-2108-703-103). The studies were conducted in accordance with the local legislation and institutional requirements. Written informed consent for participation was not required from the participants or the participants’ legal guardians/next of kin in accordance with the national legislation and institutional requirements.

## Author contributions

YW: Conceptualization, Data curation, Formal analysis, Writing – original draft, Funding acquisition. YK: Formal analysis, Methodology, Validation, Writing – original draft. SB: Conceptualization, Data curation, Investigation, Methodology, Writing – review & editing. O-KK: Methodology, Supervision, Writing – review & editing, Funding acquisition.

## Footnotes

^1^http://www.angiocalc.com

^2^https://www.r-project.org/
